# Big knowledge visualization of the COVID-19 CIDO ontology evolution

**DOI:** 10.1186/s12911-023-02184-6

**Published:** 2023-05-09

**Authors:** Ling Zheng, Yehoshua Perl, Yongqun He

**Affiliations:** 1 Computer Science and Software Engineering Department, Monmouth University, West Long Branch, NJ USA; 2 Department of Computer Science, New Jersey Institute of Technology, Newark, NJ USA; 3 Unit for Laboratory Animal Medicine, Department of Microbiology and Immunology, and Center for Computational Medicine and Bioinformatics, University of Michigan Medical School, Ann Arbor, MI USA

**Keywords:** Big knowledge visualization, COVID-19 ontology, Coronavirus ontology, CIDO ontology, Aggregate partial-area taxonomy, Summarization network, Big picture evolution, Evolution of ontologies

## Abstract

**Background:**

The extensive international research for medications and vaccines for the devastating COVID-19 pandemic requires a standard reference ontology. Among the current COVID-19 ontologies, the Coronavirus Infectious Disease Ontology (CIDO) is the largest one. Furthermore, it keeps growing very frequently. Researchers using CIDO as a reference ontology, need a quick update about the content added in a recent release to know how relevant the new concepts are to their research needs. Although CIDO is only a medium size ontology, it is still a large knowledge base posing a challenge for a user interested in obtaining the “big picture” of content changes between releases. Both a theoretical framework and a proper visualization are required to provide such a “big picture”.

**Methods:**

The child-of-based layout of the weighted aggregate partial-area taxonomy summarization network (WAT) provides a “big picture” convenient visualization of the content of an ontology. In this paper we address the “big picture” of content changes between two releases of an ontology. We introduce a new DIFF framework named Diff Weighted Aggregate Taxonomy (DWAT) to display the differences between the WATs of two releases of an ontology. We use a layered approach which consists first of a DWAT of major subjects in CIDO, and then drill down a major subject of interest in the top-level DWAT to obtain a DWAT of secondary subjects and even further refined layers.

**Results:**

A visualization of the Diff Weighted Aggregate Taxonomy is demonstrated on the CIDO ontology. The evolution of CIDO between 2020 and 2022 is demonstrated in two perspectives. Drilling down for a DWAT of secondary subject networks is also demonstrated. We illustrate how the DWAT of CIDO provides insight into its evolution.

**Conclusions:**

The new Diff Weighted Aggregate Taxonomy enables a layered approach to view the “big picture” of the changes in the content between two releases of an ontology.

## Background

The COVID-19 continues to spread all over the world with millions of casualties worldwide [1]. Scientists are continuing a frantic search for medications and vaccines for this disease with multiple presentations and recent new findings about “long COVID” – the lasting impacts in patients who recovered from COVID-19 [2]. This research requires a COVID-19 ontology for a standard to support interoperability and communication between parties. This is even more essential in view of the unprecedented level of international cooperation in the effort to find a cure and vaccines for COVID-19 [3].

Several COVID-19 ontologies have recently emerged and are available on BioPortal [4]. The Coronavirus Infectious Disease Ontology (CIDO) (10,255 concepts) [5] is created to provide a standardized representation of various coronavirus infectious diseases with the aim to support a fundamental understanding of the host-coronavirus interaction mechanisms, and to support the rational development of vaccines and drugs [6, 7]. The COVID-19 ontology (2,270 concepts) [8] covers the role of molecular and cellular entities in virus-host-interactions and in the virus life cycle, as well as medical and epidemiological concepts linked to COVID-19 [9]. The COVID-19 Infectious Disease Ontology (486 concepts) [10] is an extension of the Infectious Disease Ontology [11] and the Virus Infectious Disease Ontology [12] and covers epidemiology, classification, pathogenesis, and treatment terms used to represent infection by the SARS-CoV-2 virus strain, and the associated COVID-19 disease [13]. The WHO COVID-19 Rapid Version CRF semantic data model (398 concepts) [14], provides semantic references for the WHO COVID-19 case record form.

The largest and most quickly growing one out of the COVID-19 ontologies is the Coronavirus Infectious Disease Ontology (CIDO). Its most recent release in August 2022 has 10,255 concepts and 395 properties. As shown on BioPortal, there were 34 releases since the 1.0.06 release on January 26, 2020. These many releases are posing a comprehension problem for researchers using CIDO. One needs to know whether and to what degree the additional concepts are relevant to the user’s needs. If one concentrates on chemicals for medications, this user may want to know how many chemical concepts were added and in which categories. If a user investigates the processes in which this virus spreads, then this user would want to know how many processes were added, etc.

In this paper we will look for examples on the difference between two releases of CIDO, release 1.0.108 with 5,138 concepts published on June 14, 2020 and release 1.0.337 with 10,255 concepts published on August 1, 2022. The purpose is to provide a concise summary of the changes which will give users a “big picture” of the changes and will enable users to drill down for more details in the part(s) they are interested in, or in other words, to provide the “big picture” of the evolution of CIDO.

In previous research we developed the partial-area taxonomy summarization network [15] which provides the “big picture” of the content of an ontology. However, the partial-area taxonomy of CIDO for the above two releases contains 753 and 2,376 nodes respectively, which are too many for display, comprehension, and comparison. The weighted aggregate partial-area taxonomy (WAT) [16–18] enables to obtain a more compact summary than a partial-area taxonomy. The size of a WAT is the number of its nodes, which is controlled by a parameter. Each node of a WAT represents a major subject in the content of an ontology. In [18], we used the weighted aggregate partial-area taxonomy (WAT) to summarize the CIDO ontology (release 1.0.108). In this paper we define the Diff Weighted Aggregate Taxonomy (DWAT) between two releases of an ontology to display the differences between the WATs of the two releases. The DWAT presents the “big picture” of the evolution of the ontology from the earlier release to the latter release.

Furthermore, we will show how to “drill down” a node of interest in the top-level DWAT visually presenting meaningful changes of major subjects between releases, to further explore the “big picture” of differences in the secondary subjects summarized by the major subject node. We can even further drill down into more detailed layers.

In this paper, we introduce the DWAT summarization network and illustrate it for the CIDO ontology. However, the DWAT is applicable to display a “big picture” of the differences between two releases of any ontology.

### Area taxonomy and partial-area taxonomy

It is difficult to comprehend a large biomedical ontology representing complex domain knowledge without advanced summarization and visualization technique. Such comprehension is required in order to enhance the effective utilization of biomedical ontologies. Different summarization networks were developed by the Structural Analysis of Biomedical Ontologies Center (SABOC) [19] to summarize and visualize the “big picture” of biomedical ontologies to support ontology comprehension [15, 20, 21]. The basic building block of a summarization network is a node which summarizes a group of similar concepts. Nodes are linked by hierarchical *child-of* relationships, forming a network. Different summarization networks utilize different definitions of concept similarity.

**Fig. 1:**
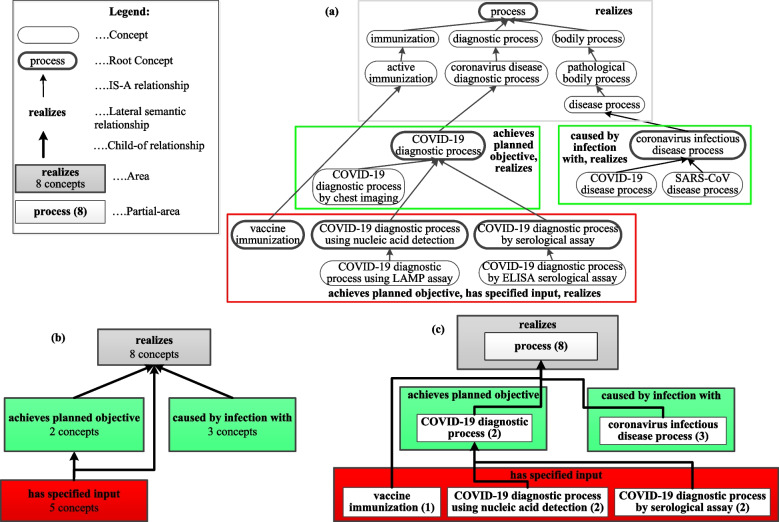
The derivation of two summarization networks for a CIDO excerpt: **a** A hierarchy excerpt of 18 process concepts from CIDO. Concepts, denoted by rounded corner boxes, are linked by hierarchical IS-A relationships represented by thin black upward arrows. Root concepts are denoted by a bold frame. Each rectangle indicates a group of concepts with exactly the same set of lateral (non-hierarchical semantic) relationship types (i.e., same structure.) Rectangles are color coded according to the number of lateral relationship types that their concepts have. The names of the lateral relationships are shown in bold inside the colored rectangles. **b** The area taxonomy summarization network for (a). The nodes, displayed as rectangular boxes filled with solid colors, represent areas and the links connecting area nodes are the hierarchical *child-of* relationships (shown as thick black upward arrows.) The area node without an emanating *child-of* link is called a root area node. Area nodes are color-coded in the same way as the colored rectangles in (a) and organized based on the length of their path to the root area node. **c** The partial-area taxonomy summarization network for (a). The partial-area taxonomy is a refinement of the area taxonomy. The nodes representing partial-areas are illustrated as white boxes within area nodes. A number in () indicates the number of concepts summarized by a partial-area. As in (b), partial-area nodes are connected by hierarchical *child-of* links shown as thick upward arrows

Two summarization networks called area taxonomy and partial-area taxonomy [22, 23] are demonstrated in Fig. 1 for an excerpt of 18 concepts from the CIDO ontology. The derivation process of the two summarization networks flows from the area taxonomy to the partial-area taxonomy which is a refinement of the former one. In Fig. 1(a), there are four color rectangles (one gray, two green, and one red), each of which includes a group of structurally similar concepts that have the same set of lateral (nonhierarchical semantic) relationship types. For example, there are eight concepts having the same lateral semantic relationship type *realizes* in the top gray rectangle. Specifically, the other seven concepts are the descendant concepts of the top concept *process*.

Concepts situated down along the hierarchy are more specific and may have new lateral relationship types in addition to those inherited from their ancestor concepts. For example, the concept *coronavirus infectious disease process* in the right green rectangle inherits the lateral relationship type *realizes* from its parent concept *disease process* in the gray rectangle and introduces one new lateral relationship type *caused by infection with*. The right green rectangle also encloses two child concepts of *coronavirus infectious disease process*, which have the same structure as their parent concept, i.e., having the same two lateral relationship types. Similarly, the red rectangle incorporates five specific process concepts having the same three lateral relationship types, one of which inherits one lateral relationship *realizes* from *active immunization* in the gray rectangle and two of which inherit *achieves planned objective* and *realizes* from *COVID-19 diagnostic process* in the left green rectangle. The third lateral relationship *has specified input* is introduced in this red rectangle.

A group of concepts with the same structure, i.e., having the same set of lateral relationship types, is defined as an area*.* Every colored rectangle in Fig. 1(a) turns into one area node in Fig. 1(b). An area node, summarizing the concepts of its area, is labeled by the set of newly introduced lateral relationship types of all its concepts and the number of concepts it summarizes. The lateral relationships inherited from ancestor nodes are not shown since they can be derived tracing back the hierarchy. For example, the top eight concepts enclosed in the gray rectangle in Fig. 1(a) are summarized by the gray area node labeled as ‘realizes—8 concepts’ in Fig. 1(b). Similarly, the three concepts in the right green rectangle are summarized by the right green area node labeled as ‘caused by infection with—3 concepts,’ since only the relationship *caused by infection with* is new for this area. Area nodes are color coded based on the total number of lateral relationship types that their concepts have (including the inherited relationships) and area nodes with the same total number of lateral relationship types have the same color. For example, both the left and the right green area nodes have a total of two lateral relationship types, although not the same two.

Six concepts in Fig. 1(a), different from others, have a bold border. These concepts are called roots of their areas because they have no parent concepts within their areas. An area may have multiple roots. For example, the red area has three roots, *vaccine immunization*, *COVID-19 diagnostic process using nucleic acid detection* and *COVID-19 diagnostic process by serological assay* having different semantics. The hierarchical *child-of* links connecting area nodes are defined according to the hierarchical IS-A relationships emanating from area roots in the underlying ontology. An area *A* is *child-of* an area *B* if a root in *A* has a parent in *B*. For example, the red area node is *child-of* both the gray area node and the left green area node in Fig. 1(b), since the root *vaccine immunization* in the red area is a child concept of *active immunization* in the gray area and the other two roots are children of the root *COVID-19 diagnostic process* in the left green area. The area taxonomy is a network consisting of the area nodes and the *child-of* links connecting them [22]*.*

Although the area taxonomy, having only four area nodes in Fig. 1(b), provides a compact summary of the ontology excerpt in Fig. 1(a), Fig. 1(b) is derived based only on the structure (i.e., lateral relationships) without utilizing concept semantics. For example, the three root concepts in the red rectangle in Fig. 1(a) have different semantics as reflected by their names, although they have the same set of three lateral relationship types (i.e., the same structure). Hence, to further refine the area taxonomy, an area is divided into smaller units called partial-areas, each of which consists of one root concept and all its descendant concepts in the same area. The concepts of a partial-area share similar semantics, since they inherit the semantics of the root of the partial-area.

A partial-area is denoted as a white partial-area node within an area node in Fig. 1(c), and is labeled with the name of the root concept, representing its semantics, followed by the number of all concepts in this partial-area inside (). For example, the root *COVID-19 diagnostic process using nucleic acid detection* and its child concept in the red area of Fig. 1(a) have similar semantics, so they are summarized in Fig. 1(c) by the partial-area node ‘COVID-19 diagnostic process using nucleic acid detection (2)’. Partial-area nodes are also connected by *child-of* links (defined similar to those in the area taxonomy), forming the partial-area taxonomy (PAT) [23]. The partial-area taxonomy summarization network in Fig. 1(c) captures both the structural and semantic information of Fig. 1(a). It includes six partial-area nodes within four area nodes, and provides a better summary of Fig. 1(a) with more details than Fig. 1(b).

### Child-of-based layout of an aggregate taxonomy

A partial-area taxonomy may provide a “big picture” view of a small ontology. However, for a large ontology this summarization network may still be too overwhelming to be easily comprehensible. For example, the latest release (release 1.0.337) of CIDO involved in this research has 10,255 concepts. Its partial-area taxonomy has 409 area nodes and 2,376 partial-area nodes. Hence, both the area taxonomy and the partial-area taxonomy are too large for visualization and comprehension. To provide a more comprehensible view of such an ontology, the weighted aggregate partial-area taxonomy (Weighted Aggregate Taxonomy, WAT for short) was developed [16–18]. It introduces a user-specified integer parameter *b* to control which nodes of a partial-area taxonomy (PAT) will be shown.

The weight of a partial-area is defined as the number of the descendant concepts of its root 'r' in the whole ontology, not only considering the concepts in the partial-area 'r' itself, but also the concepts in all its descendant partial-areas, since they are also semantic refinements of 'r.' In a WAT, only partial-areas with a weight ≥ b, considered as large, are shown as nodes representing major subjects in an ontology. However, partial-areas with a weight less than *b*, considered as small, are not lost. They are aggregated into their closest large ancestor partial-area(s) with a weight ≥ b. To control the size of the WAT, users can vary the parameter *b*. Note, the aggregated small partial-areas can be displayed again using a ‘drill down’ process [16], when needed, utilizing the Ontology Abstraction Framework (OAF) software [24], which is available at [25].

In a WAT, there are two types of partial-area nodes. One is partial-area nodes not aggregating any descendant partial-area, which are shown as they are in a PAT. The other is aggregate partial-area nodes, aggregating small descendant partial-areas, which are displayed as rounded corner rectangles and are labeled by the name of their original root followed by three numbers enclosed in (),{}, and [], respectively. The first number in () is the total number of concepts in the aggregate partial-area node after aggregation, the second one in {} means the number of descendant partial-areas which were aggregated into this aggregate partial-area node. The last one in [] is the original number of concepts in this partial-area before aggregation.

Furthermore, the new child-of-based layout of an aggregate taxonomy introduced in [18], arranges (aggregate) partial-areas according to the length of child-of paths to the root (aggregate) partial-area. In other words, the partial-areas, having the same length of their child-of path to the root partial-area, are at the same level.

**Fig. 2:**
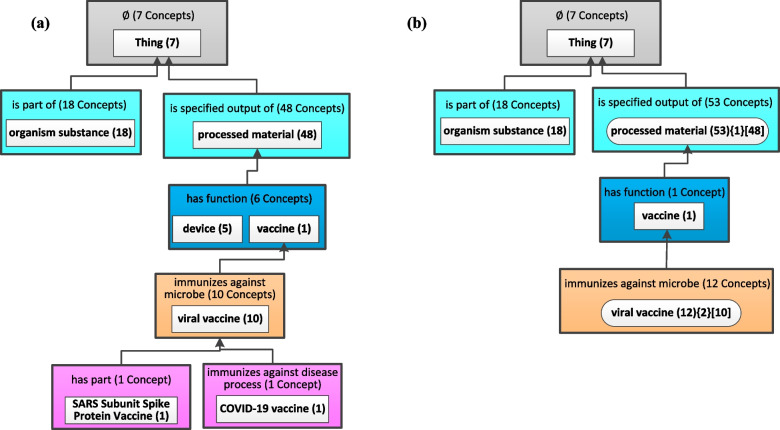
The aggregation process of an excerpt of the CIDO partial-area taxonomy: **a** An excerpt of the partial-area taxonomy for the June 14, 2020 release of CIDO. **b** The weighted aggregate taxonomy obtained for the partial-area taxonomy in (a) using parameter *b* = 10. The aggregate partial-area nodes are shown with rounded-corner rectangles

Figure 2(a) shows an excerpt of the partial-area taxonomy for the June 14, 2020 release of CIDO. Given the parameter *b* for the WAT, as 10, the middle partial-area labeled as ‘device (5)’ and the bottom two partial-areas, each with a single concept, are considered small, since they have no descendant partial-areas and their weight is the same as the number of concepts they summarized, which is less than *b*. They are aggregated into their closest large ancestor partial-area. The aggregation results in two aggregate partial-areas displayed in Fig. 2(b) as a rounded corner rectangle. They are ‘processed material (53){1}[48]’ and ‘viral vaccine (12){2}[10]’. Consider the latter one for example, the last number 10 is the original number of concepts in the partial-area ‘viral vaccine’ before the aggregation, as seen in Fig. 2(a). The first number 12 means this aggregate partial-area now summarizes 12 concepts since two concepts from its small child partial-areas are aggregated into it. The second number 2 means this aggregate partial-area has two of its small partial-areas, in Fig. 2(a), aggregated into it.

All other partial-areas in Fig. 2(a) have a weight greater than 10. In addition, they have no small descendant partial-areas to be aggregated to them. Hence, they are shown in Fig. 2(b), as regular partial-areas, the same as they are in Fig. 2(a). Note, although the number of concepts summarized by the partial-area ‘vaccine (1)’ is 1, its weight is 13 (= 1 + 10 + 1 + 1) counting the number of concepts in its three descendant partial-areas. Thus, it is a large partial-area given *b* = 10. Similarly the root partial-area ‘Thing (7)’ has a weight greater than 10, since all concepts in Fig. 2(a) are the descendants of the root concept ‘Thing.’

## Methods

Given two releases of an ontology and their weighted aggregate taxonomy, we define a new summarization network called Diff Weighted Aggregate Taxonomy (DWAT), to summarize and visualize the “big picture” of content changes between the two releases. The DWAT enables an easy comprehension of the ontology evolution. This new Diff summarization network reflects the comparison of major subjects at the top-level and their content between two releases. In contrast, the two Diff summarization networks previously developed by Ochs et al. [26], the Diff Area Taxonomy and the Diff Partial-area Taxonomy (DPAT), are focused on the difference between two releases of the area and partial-area taxonomies, respectively. These two former Diff summarization networks reflect changes of concepts with regard to an area or a partial-area. They enable visualization changes between two releases of small ontologies or of small excerpts of large ontologies. However, they cannot visualize the difference between two releases of an even medium size ontology like CIDO as a partial-area taxonomy cannot comprehensibly visualize CIDO, since the partial-area taxonomy for the most recent release of CIDO has 409 areas and 2,376 partial-areas. For visualizing a summary of CIDO a weighted aggregate partial-area taxonomy is required [18]. Thus, we need to define DWAT for visualizing the difference between releases of CIDO or other medium size or large ontologies.

The inputs of a DWAT are two weighted aggregate taxonomies T_1_ and T_2_ derived from two different releases O_1_ and O_2_ of an ontology O (O_1_ is an older one). A DWAT is composed of *diff nodes* connected by *child-of* relationships. The derivation of a DWAT starts by a top-down comparison between the two sets of major subjects (i.e., nodes) in T_1_ and T_2_ and their contents. There are four types of *diff nodes* in a DWAT, as follows.

- An *Introduced Node* indicates a new major subject appearing in T_2_ but not in T_1_. Such a node may contain newly added concepts in O_2_, or contain existing concepts in O_1_ but with changes resulting in such new node in T_2_. An introduced node is visualized with a green background for the partial-area with the same shape (rectangle or rounded corner rectangle) and label as in T_2_.
- A *Removed Node* indicates a major subject appearing in T_1_ but not in T_2_. This may be due to concepts in such a node of T_1_ which no longer exist in O_2_. Another possibility is that concepts in such a node of T_1_ also exist in O_2_, but with changes thus appearing in another node in T_2_. T_2_ may also use a larger *b* than T_1_, then a node in T_1_ may not qualify for a node in T_2_. In such a case the concepts of this node will be aggregated into an ancestor node. A removed node is visualized with a red background for the partial-area with the same shape and label as in T_1_.
- A *Modified Node* indicates a major subject appearing in both T_1_ and T_2_ but the summarized sets of concepts of the major subject are not identical. This may be due to newly added concepts in O_2_, or concepts removed from O_2_, and/or changes of existing concepts resulting in concepts moved in or out of this node in T_2_. A modified node is visualized with a yellow background for the partial-area with the same shape and label as in T_1_.
- An *Unmodified Node* is a node which appears in both T_1_ and T_2_ and summarizes the same set of concepts in both T_1_ and T_2_. The background of the partial-area which is an unmodified node stays white.

We note that the DWAT is considerably more complex than the Diff Partial-area Taxonomy (DPAT) described in [26]. While the changes recorded in DPAT were in the number of concepts, in the DWAT we need to record changes in the number of concepts, partial-areas and relationships. The information in the OAF tool [24] enables to calculate the changes in the number of concepts added, removed or modified for each diff node of DWAT.

Correspondingly, if a *child-of* relationship connecting two major subject nodes exists in T_2_ but not in T_1_, then it is an *introduced child-of* marked as a green arrow*.* If a *child-of* relationship is in T_1_ but not T_2_, then it is called a *removed child-of* marked as a red arrow*.* An *unmodified child-of* relationship connects the same two nodes in both T_1_ and T_2_. Note that there is no *modified child-of* relationship, since a relationship either exists or not.

**Fig. 3:**
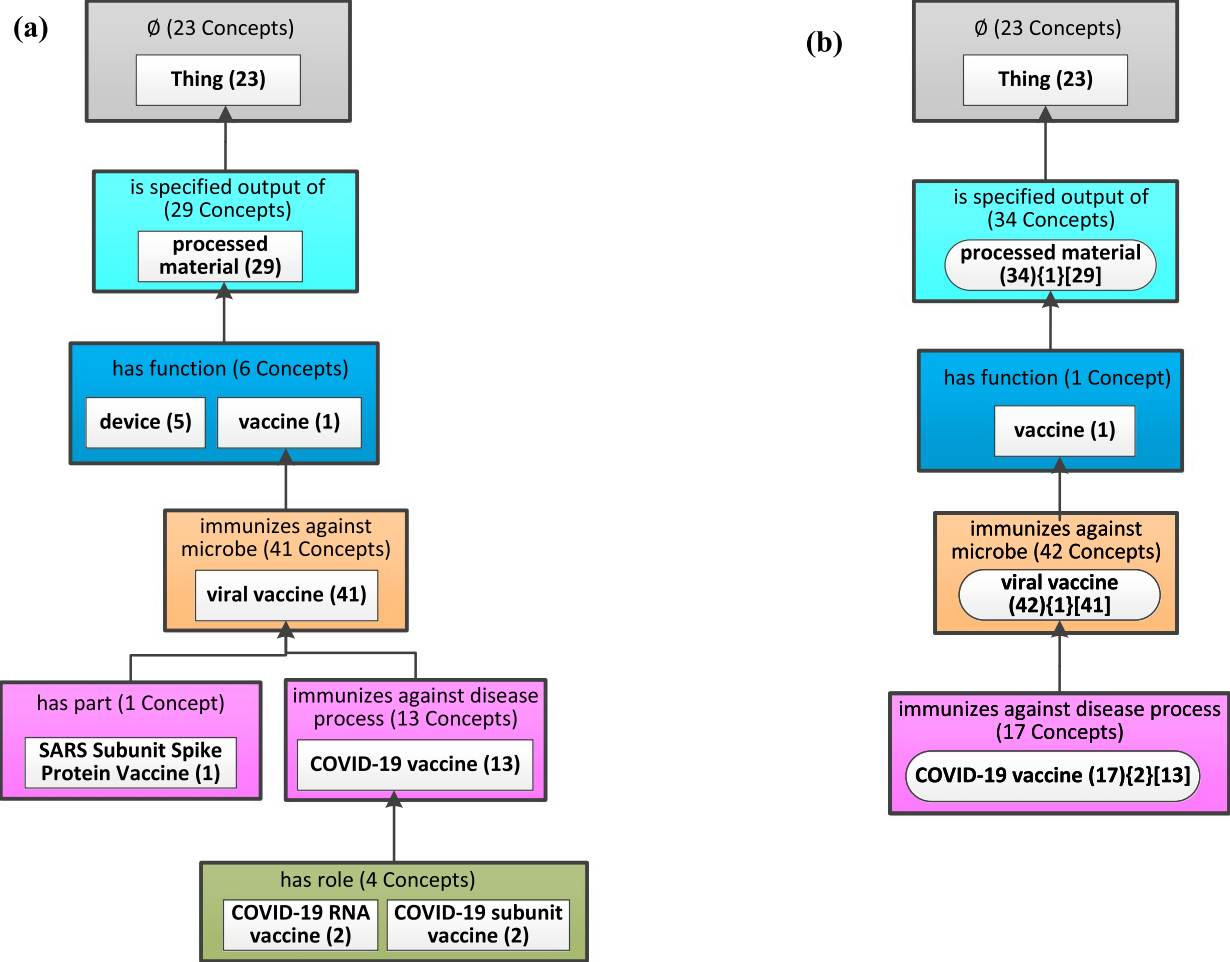
**a** The excerpt of the partial-area taxonomy for the August 1, 2022 release of CIDO, corresponding to Fig. 2. **b** The weighted aggregate taxonomy obtained for (a) using *b* = 10

Figure 3 shows the derivation of the weighted aggregate taxonomy from the excerpt of the partial-area taxonomy rooted at *Thing* for the August 1, 2022 release of CIDO, corresponding to Fig. 2. Here the parameter *b* = 10 is the same as in Fig. 2. Note that in the newer release, the partial-area ‘COVID-19 vaccine’ has more descendant concepts and it has two child partial-areas which are small having a weight less than *b* = 10. Hence, the two child partial-areas at the bottom of Fig. 3(a) are aggregated into the partial-area ‘COVID-19 vaccine’, resulting in the bottom aggregate partial-area in Fig. 3(b). The derivation of the other two aggregate partial-areas rooted at *processed material* and *viral vaccine* is the same as Fig. 2.

**Fig. 4:**
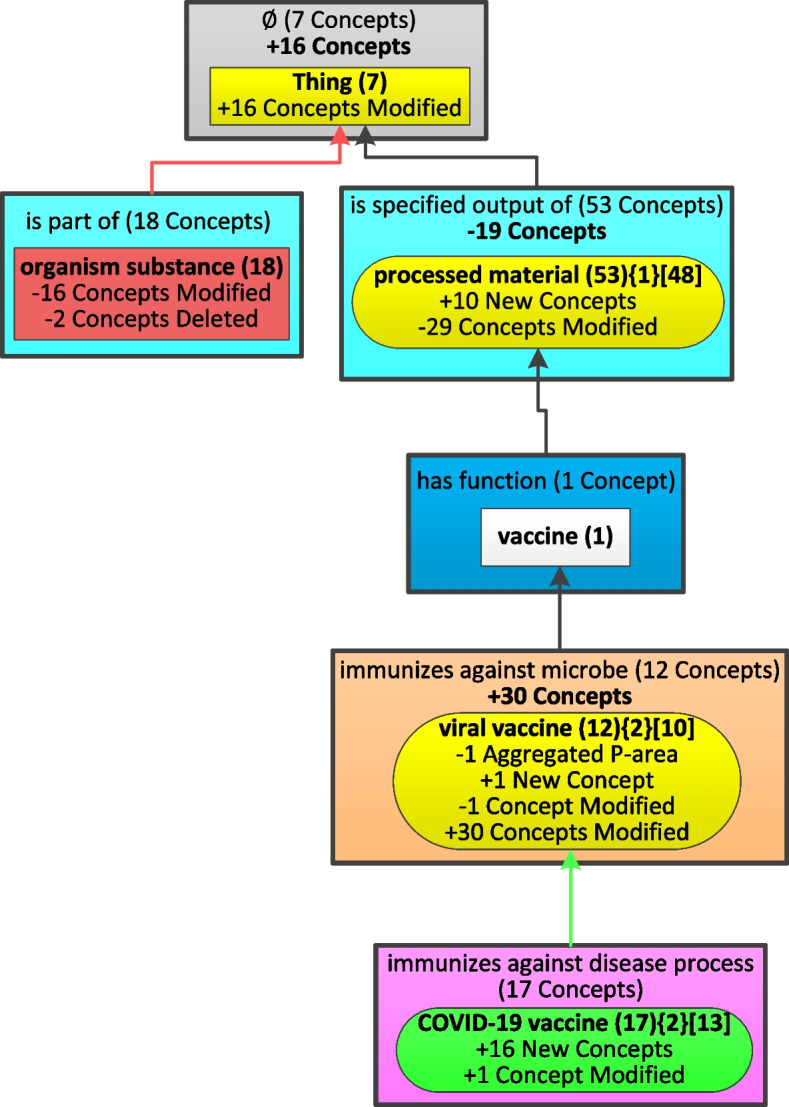
The Diff Weighted Aggregate Taxonomy between the two WATs of Figs. 2(b) and 3(b). Modified nodes, unmodified nodes, introduced nodes, and removed nodes are shown with a yellow, white, green, and red background, respectively. *Child-of* relationships connecting diff nodes are colored green, red, or black if they were introduced, removed, or unmodified, respectively. A summary of content changes is shown below the label of each area and partial-area. For the areas containing diff nodes, the global changes of the number of relationships and concepts are shown in bold below the label of such areas. To avoid information redundancy, we do not show such information for areas which have changes all due to introduced nodes or removed nodes, since it is obvious that all different concepts are new or removed from this area, respectively

Figure 4 shows how to derive the Diff Weighted Aggregate Taxonomy summarizing the differences between the two WATs in Figs. 2(b) and 3(b). The comparison progresses top-down illustrating various situations with specific nodes. We start to describe the changes at the area level and then continue at the partial-area level. The former kind of changes is global since an area may have multiple partial-areas while the changes in a partial-area are local, just for this unit. When displaying the labels of the area and partial-area nodes in the DWAT for two taxonomy versions T_1_ and T_2_, we present the labels from T_1_ which is the base WAT for the changes described by the DWAT unless it is an introduced node which does not exist in T_1_. We note that in the following examples, areas have only one partial-area, which is just a coincidence. Examples with multiple partial-areas in an area will appear in the Results section.

The top partial-area ‘Thing’, summarizing concepts without relationships, appears in both Fig. 2(b) and Fig. 3(b), but it summarizes different number of concepts, 7 and 23 respectively. It is the only partial-area in its area. Hence, for the changes regarding its area, the number of concepts increased from 7 to 23 is represented as ‘** + 16 Concepts**’ in bold under the label of the area which is the same as in the older release (Fig. 2(b)). For the local changes regarding the ‘Thing’ partial-area node shown in Fig. 4, it has the same shape and label as in the older base Fig. 2(b). Those additional 16 concepts are existing concepts in the old release but now have no relationship in the most recent release. Such a change is shown as ‘ + 16 Concepts Modified’. The symbol ‘ + ’ means the increasing number of concepts in this node. Hence, the node ‘Thing’ is a Modified Node with a yellow background in Fig. 4.

We are moving down to the second level under the root node. Figure 2(b) has two nodes ‘organism substance (18)’ and ‘processed material (53){1}[48]’, while the former one does not exist in Fig. 3(b). Hence, ‘organism substance (18)’ should be shown as a Removed Node with a red background in Fig. 4. The change is due to two concepts which do not exist anymore, and the other 16 concepts which do not have any relationships in the most recent release, and thus were moved to the root node. Hence, ‘-16 Concepts Modified’ and ‘-2 Concepts Deleted’ are shown under the partial-area label ‘organism substance (18).’ The symbol ‘-’ means a decreasing number of concepts in this node. Its area contains only the node itself, so the changes of its area are obvious, i.e., the number of relationships and the number of concepts reduce to 0, and are omitted at the area level to avoid redundancy. The child-of link from this removed node to the root node is a removed child-of shown as a red arrow.

The second node rooted at *processed material* in Fig. 2(b) summarizes 53 concepts including its original 48 concepts before the aggregation and 5 concepts aggregated from its child small partial-area. This node is also shown in Fig. 3(b) but it summarizes 34 concepts and aggregates the same small partial-area. The two areas containing this node have the same relationship, hence, there is no change in the listed relationships. There is a reduction of 19 concepts at the area level from 53 to 34. Hence, the global change shown in Fig. 4 regarding the area is ‘**-19 Concepts**’ under the area label. The partial-area has the same shape and label as in the older base Fig. 2(b). Regarding the local changes, the change in the number of concepts is due to 10 concepts added in the newer release (shown as ‘ + 10 New Concepts’) and the modeling change of 29 concepts which are moved out from this partial-area (shown as ‘-29 Concepts Modified’.) We see that this node ‘processed material’ appears in Fig. 4 as a Modified Node with a yellow background. The child-of link from ‘processed material’ to ‘Thing’ exists in both figures, hence, it is an unmodified child-of relationship shown as a black upward arrow.

The node ‘vaccine (1)’ appears the same in Fig. 2(b) and Fig. 3(b) and resides in the same area in both figures. Hence, there is no change at the area level and this node is an Unmodified Node with a white background. Similarly, the child-of link pointing to ‘processed material’ exists in both figures, and hence is an unmodified one.

It is clear that the node ‘viral vaccine’ should be a Modified Node in Fig. 4 since it exists in the same area in both Fig. 2(b) and Fig. 3(b). However, it summarizes different number of concepts, 12 and 42 respectively. Since this node is the only one inside its area, the change regarding the area is the number of concepts shown as ‘ **+ 30 Concepts**’ in bold under the label of the area. Comparing the node ‘viral vaccine’ in Fig. 2(b) and Fig. 3(b), we can see that the number of aggregated partial-areas reduces from two to one, which is shown as ‘-1 Aggregated P-area’ under the label of the partial-area. The reduction is due to ‘COVID-19 vaccine’ becoming an aggregate partial-area in Fig. 3(b). This results from more COVID-19 vaccine concepts in the newer release of CIDO, making the node ‘COVID-19 vaccine’ itself a major subject in the WAT with *b* = 10. In addition, we find that there is one new concept (shown as ‘ + 1 New Concept’) in the newer release and 31 concepts having changes in their modeling including one moved out from this node (shown as ‘-1 Concept Modified’) and 30 concepts moved into this node from others (shown as ‘ + 30 Concepts Modified’). Similarly, the child-of link pointing to ‘vaccine’ is unmodified.

Since the node ‘COVID-19 vaccine’ does not exist in Fig. 2(b) at all but appears in Fig. 3(b), it is an Introduced Node with a green background in Fig. 4. The node has the same label as in the WAT in Fig. 3(b). Checking the 17 concepts summarized by the ‘COVID-19 vaccine’ node in the two releases, we see that 16 concepts are new (shown as ‘ + 16 New Concepts’) and the modeling of one existing concept *COVID-19 vaccine* which is aggregated into ‘viral vaccine’ in Fig. 2(b) is now modified (shown as ‘ + 1 Concept Modified’). The child-of link connecting this node to ‘viral vaccine’ is an introduced link shown as a green upward arrow. If all the changes of an area are due to introduced nodes or removed ones, the changes of the area are omitted to avoid information redundancy with the changes listed for the partial-areas. For example, the area containing the introduced node ‘COVID-19 vaccine’ has only one partial-area. Thus, the change of the area is the same as the change of the introduced node.

Each node in a WAT representing a major subject of an ontology can be expanded into a secondary subject taxonomy using the OAF tool to show the aggregated partial-areas under the major subject node [18]. If one is interested in more detailed view of the evolution of a major subject represented by a node in a DWAT, then this can be done by drilling down this major subject to obtain the DWAT for the two secondary subject taxonomies under this major subject in two releases. Illustration with examples appears in the Results section.

## Results

### Evolution of CIDO major subjects by the Diff Weighted Aggregate Taxonomy

**Fig. 5:**
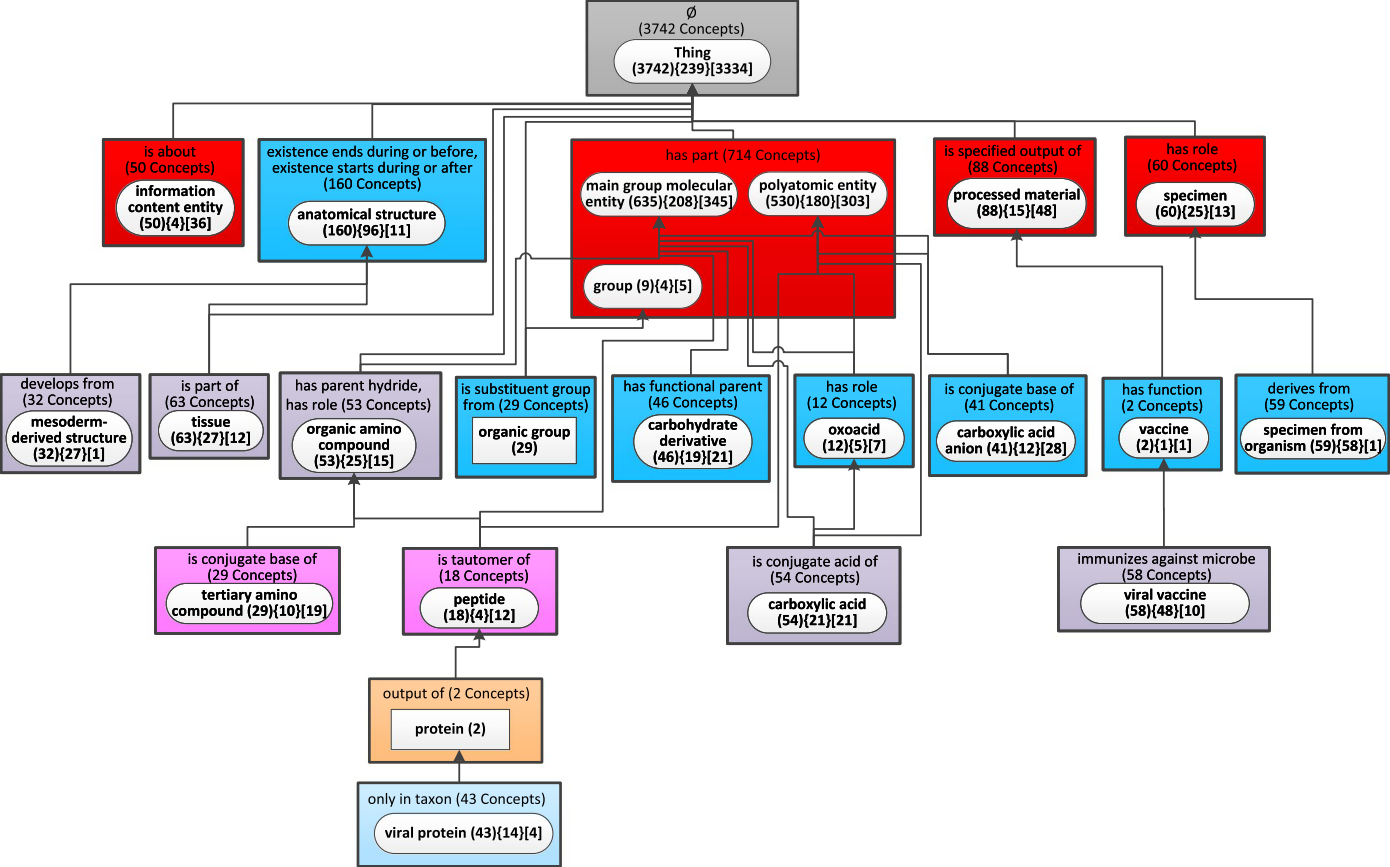
The weighted aggregate taxonomy for the June 2020 release of CIDO using *b* = 29

**Fig. 6:**
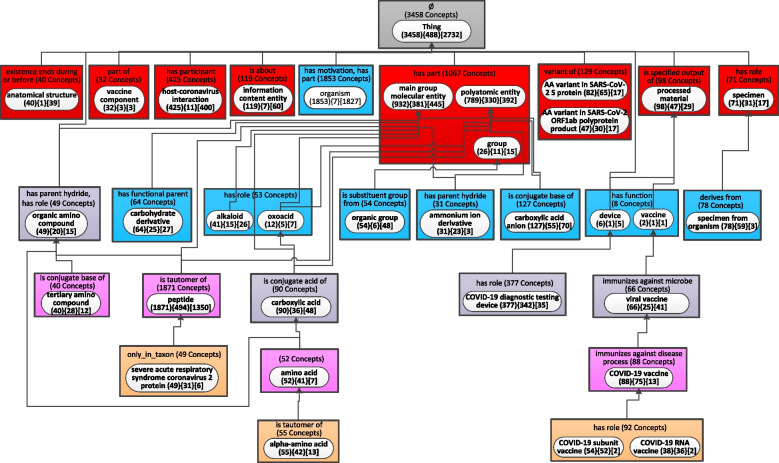
The weighted aggregate taxonomy for the August 2022 release of CIDO using *b* = 29 same as the value of *b* for the old release in Fig. 5

**Fig. 7:**
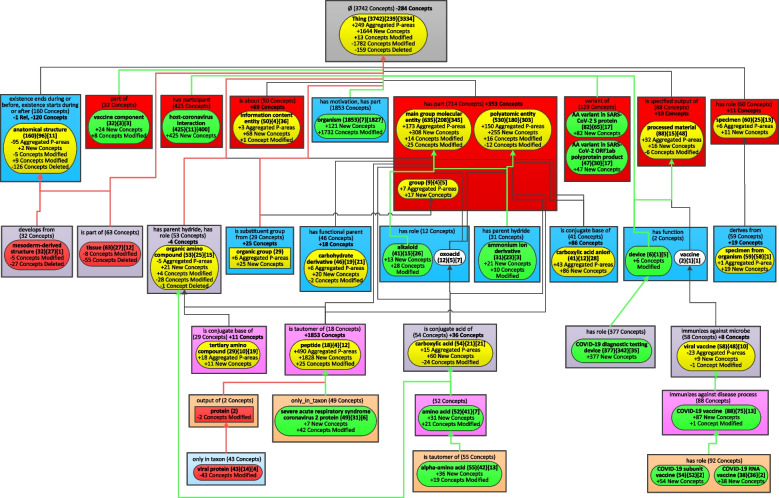
The Diff Weighted Aggregate Taxonomy between the two WATs of Figs. 5 and 6

To show the evolution of the CIDO ontology, the weighted aggregated taxonomy (WAT) with the parameter b = 29 (Fig. 5) generated for release 1.0.108 published on June 14, 2020, and the WAT with the same b = 29 (Fig. 6) for the most recent release 1.0.337 published on August 1, 2022, are used to derive the Diff Weighted Aggregate Taxonomy (DWAT) (Fig. 7). The b value 29 is the smallest integer generating the WAT for the older release with at most 25 major subject nodes. Such WATs with up to 25 nodes are comfortable for human to comprehend the “big picture” of an ontology [16]. Figure 5 has 23 nodes while Fig. 6 has 34 nodes since more concepts were added meanwhile to CIDO.

The most recent release of CIDO has 10,255 concepts which is almost twice of the 5,138 concepts in the June 2020 release. It is impossible to capture the “big picture” of changes at such magnitude without summarization techniques [15]. Figure 7 summarizes the changes of CIDO from June 2020 to August 2022 while the knowledge of COVID-19 has been enhanced in these last two years.

There are 15 introduced nodes in Fig. 7 with green solid background representing new major subjects of CIDO which did not exist in 2020. These nodes are just in Fig. 6 but not in Fig. 5. Six of them become major subjects due to newly added concepts in the most recent release. They are: ‘host-coronavirus interaction’ (425 new concepts), ‘AA variant in SARS-CoV-2 S protein’ (82), and ‘AA variant in SARS-CoV-2 ORF1ab polyprotein product’ (47) at the second level inside red rectangles, ‘COVID-19 diagnostic testing device’ (377) at the fourth level, ‘COVID-19 subunit vaccine’ (54), and ‘COVID-19 RNA vaccine’ (38) at the bottom.

The other eight green nodes ‘vaccine component’, ‘organism’, ‘alkaloid’, ‘ammonium ion derivative’, ‘severe acute respiratory syndrome coronavirus 2 protein’, ‘amino acid’, ‘alpha-amino acid’, and ‘COVID-19 vaccine’ become new major subjects due to both new concepts and changes in the existing concepts. For example, in the old release, there was only one concept *COVID-19 vaccine* which was summarized by the major subject ‘viral vaccine.’ However, the node ‘COVID-19 vaccine’ has a weight of 180 (greater than *b* = 29) making it a major subject in the most recent release, which is due to 179 new COVID-19 vaccine related concepts added in the most recent release including 87 concepts in the ‘COVID-19 vaccine’ node (shown as ‘ + 87 New Concepts’) and 92 concepts in its two child partial-areas. The notation ‘ + 1 Concept Modified’ in the node ‘COVID-19 vaccine’ means that one concept, i.e., COVID-19 vaccine, was moved into this node from another one due to changes.

The green node ‘device’ appears as a major subject in the new release due to its child node ‘COVID-19 diagnostic testing device’ increasing the weight of ‘device’ to 383 (= 6 + 377) greater than b = 29. The new concepts represented by these introduced nodes are discussed in the recent CIDO paper which presented a comprehensive update on CIDO [27].

Four major subjects in the old release disappear from the most recent release, represented as removed nodes shown with red background. The two nodes ‘protein’ and ‘viral protein’ are due to the modeling changes of existing concepts in the new release. The other two nodes are ‘mesoderm-derived structure’ and ‘tissue’. These two root concepts are summarized now by the node ‘anatomical structure’ due to the modeling change in the new release, which is respectively reflected as ‘-5 Concepts Modified’ and ‘-8 Concepts Modified’ in the two nodes. Furthermore, the other concepts in these two nodes are no longer in the most recent release, which is reflected as ‘-27 Concepts Deleted’ and ‘-55 Concepts Deleted’ in the two nodes.

There are 17 nodes appearing in both releases as major subjects (in both Figs. 5 and 6) but summarizing different sets of concepts. They are modified nodes which are highlighted in yellow in Fig. 7. For example, 25 new concepts in the new release are summarized by the node ‘organic group’, which is reflected as ‘ + 25 New Concepts’. These additions increase the number of aggregated partial-areas from 0 to 6 (‘ + 6 Aggregated P-areas’). Hence, the node ‘organic group’ is an aggregate partial-area shown differently as a rounded-corner rectangle in Fig. 6 rather than a regular rectangle in Fig. 5. We also noted that there is no longer an is-a relationship from the two root concepts *organic group* and *organic amino compound* to the concept *molecular entity* in the root node ‘Thing’ in the most recent release. Hence, there is no longer a child-of relationship from the two nodes ‘organic group’ and ‘organic amino compound’ to the root node. Those are removed child-of relationships shown as a red arrow in Fig. 7.

Figure 7 has only one area with changes in terms of relationship types, where the node ‘anatomical structure’ is. This node in the old release (Fig. 5) had two relationship types one of which was removed in the new release (Fig. 6). Hence, the notation ‘-1 Rel’ in bold is shown under the area label. Other areas have only differences in the number of concepts, e.g., the root area has the notation ‘-284 Concepts’ indicating there is a reduction of 284 concepts without relationships from the old release to the new release. The two nodes, ‘oxoacid (12){5}[7]’ and ‘vaccine (2){1}[1]’, stay the same in both releases. They are unmodified nodes shown as white in Fig. 7.

**Fig. 8:**
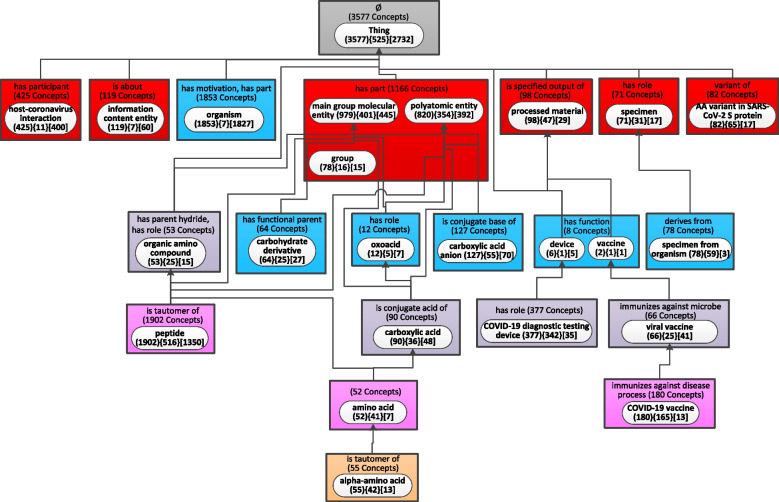
The weighted aggregate taxonomy for the August 2022 release of CIDO using *b* = 55

**Fig. 9:**
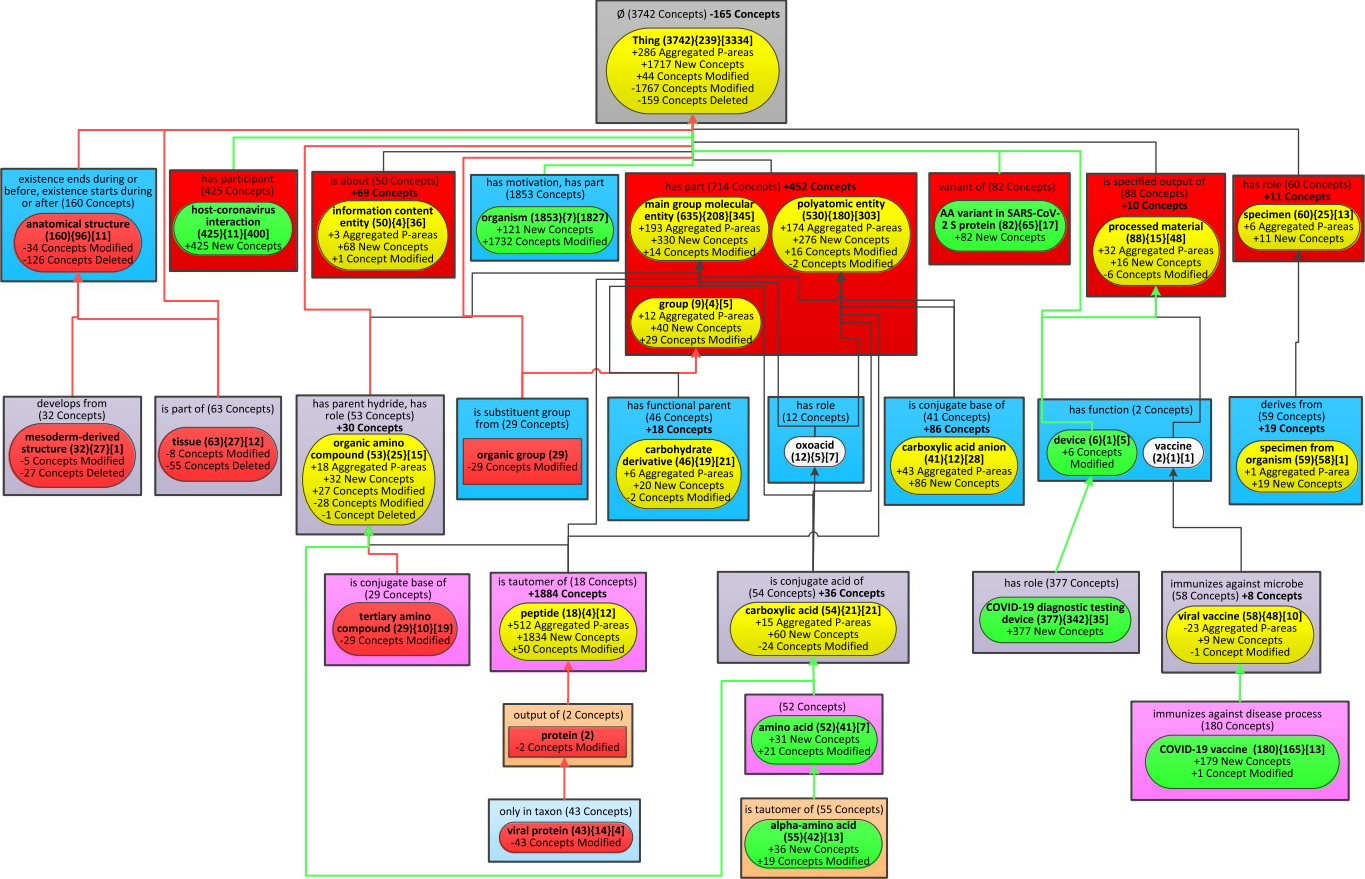
The Diff Weighted Aggregate Taxonomy between the two WATs of Figs. 5 and 8

The WAT for the new release of CIDO in Fig. 6 with more than 25 nodes was derived using the parameter b = 29 which is the same parameter that makes the WAT for the old release have at most 25 nodes. According to our previous research [16], it is preferred for “big picture” comprehension of an ontology to have a WAT with at most 25 nodes. Figure 8 shows the WAT for the new release of CIDO with 24 nodes using b = 55. Since the value of b is larger than the parameter 29 for Fig. 6, some nodes in Fig. 6 are further aggregated and do not appear in Fig. 8. We also derived the Diff Weighted Aggregate Taxonomy (Fig. 9) based on the WAT with b = 29 in Fig. 5 for the old release and the WAT with b = 55 in Fig. 8 for the most recent release.

There are eight introduced green-highlighted nodes in Fig. 9, rather than 15 in Fig. 7. Seven of these eight nodes have exactly the same Diff information as in Fig. 7. Because these nodes in Fig. 8 have the same information as in Fig. 6, since increasing b does not affect these nodes. They are ‘host-coronavirus interaction’, ‘organism’, ‘AA variant in SARS-CoV-2 S protein’, ‘device’, ‘COVID-19 diagnostic testing device’, ‘amino acid’, and ‘alpha-amino acid.’ However, increasing b makes the two nodes ‘COVID-19 subunit vaccine’ with a weight 54 and ‘COVID-19 RNA vaccine’ with a weight 38, shown at the bottom in Fig. 6, considered as small nodes and they are aggregated into their parent node ‘COVID-19 vaccine’ in Fig. 8. Hence, the introduced green node ‘COVID-19 vaccine’ in Fig. 9 has a total of 180 concepts including 179 new concepts (‘ + 179 New Concepts’) and the concept *COVID-19 vaccine* moving from the node ‘viral vaccine’ in the old release (reflected as ‘ + 1 Concept Modified’).

While there are less introduced nodes in Fig. 9 than in Fig. 7, the number of removed nodes increases in Fig. 9 due to the larger b. Figure 9 has seven removed nodes, with red background. They appear as major subjects in Fig. 5 but disappear from Fig. 8. Four of them, i.e., ‘mesoderm-derived structure’, ‘tissue’, ‘protein’, and ‘viral protein’, have the same diff information as in Fig. 7. From Fig. 7, we can see that there is an increase of concepts related to ‘organic group’ and ‘tertiary amino compound’ in the new release. However, they still have less than 55 descendant concepts, causing them to disappear from the WAT in Fig. 8. Thus, each of these two nodes has the notation ‘-29 Concepts Modified’ in Fig. 9.

The node ‘anatomical structure’ has a weight 40 in the new release in Fig. 6, which is less than b = 55. Thus, it is not a major subject in Fig. 8. Hence, it is a removed node in Fig. 9. Checking the 160 concepts in the node ‘anatomical structure’ in the old release, we found that 126 are removed from the new release (‘-126 Concepts Deleted’) and other 34 concepts are aggregated into the root node (‘-34 Concepts Modified’).

There are only 14 modified nodes highlighted in yellow in Fig. 9. For example, the 29 concepts summarized by the node ‘organic group’ in the old release were aggregated into its parent node ‘group’ in the new release. This modification is reflected as ‘ + 29 Concepts Modified’ in the yellow modified node ‘group.’ In addition, the node ‘group’ has 40 newly added concepts in the most recent release (‘ + 40 New Concepts’). These additions increase the number of aggregated partial-areas by 12 (‘ + 12 Aggregated P-areas’). Similar as in Fig. 7, the child-of link from the node ‘organic amino compound’ to the root node ‘Thing’ is a removed child-of shown as a red arrow in Fig. 9. The two major subject nodes ‘oxoacid (12){5}[7]’ and ‘vaccine (2){1}[1]’ are unmodified nodes in Fig. 9, as in Fig. 7.

### Evolution of secondary subjects in CIDO by the Diff Weighted Aggregate Taxonomy

Figures 7 and 9 capture the changes of the content in the top-level major subjects between two releases of CIDO. The WAT as the first layer is also called a major subject network where each node represents a major subject of CIDO. When users are interested in a specific major subject, they can drill down into it to get more details. The OAF tool [24] can generate a secondary subject network displaying the hidden small partial-areas that were aggregated into this major subject node. For example, we can drill down into the major subject node ‘peptide (1871){494}[1350]’ in Fig. 6 to get a secondary subject taxonomy consisting of the original node ‘peptide’ without aggregation summarizing 1350 concepts and its 494 small descendant nodes. To control the number of nodes for easy comprehension, a secondary subject taxonomy is also a WAT obtained by applying aggregation. From Fig. 7, we can see that between the two releases, the major subject node ‘peptide’ has gone through an explosive change. To show the evolution of the secondary subjects under the major subject ‘peptide’, we will derive a DWAT between two secondary subject taxonomies.

**Fig. 10:**
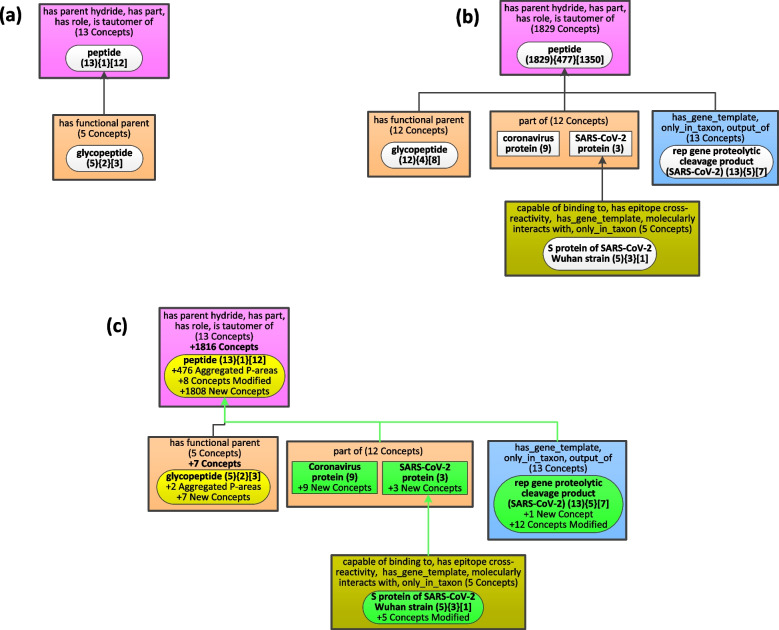
**a** the secondary subject WAT (b = 5) under the major subject ‘peptide’ in Fig. 5. **b** the secondary subject WAT (b = 5) under the major subject ‘peptide’ in Fig. 6. **c** the Diff Weighted Aggregate Taxonomy between the two WATs of (a) and (b)

Figure 10(a) shows the WAT with the parameter b = 5 for the secondary subject taxonomy of the major subject node ‘peptide (18){4}[12]’ in Fig. 5. The WAT with the same b = 5 for the secondary subject taxonomy of the major subject node ‘peptide (1871){494}[1350]’ in Fig. 6 is shown in Fig. 10(b). Figure 10(c) shows the Diff Weighted Aggregate Taxonomy between the two WATs to capture the evolution of the secondary subjects under the major subject ‘peptide’. We can see that four secondary subject nodes are new and highlighted in green. Two of them, i.e., ‘Coronavirus protein’ node with 9 concepts and ‘SARS-CoV-2 protein’ node with 3 concepts, are due to their newly added concepts in the new release. The five concepts in the ‘S protein of SARS-CoV-2 Wuhan strain’ node are existing concepts from the old release with modeling changes in the new release. The node ‘rep gene proteolytic cleavage product (SARS-CoV-2)’ is due to one new concept and modeling changes of 12 existing concepts.

The secondary subject node ‘glycopeptide’ is a modified node with seven new concepts added in the new release. The major changes are in the modified secondary major node ‘peptide’ due to 1808 newly added concepts and eight existing concepts with modeling changes. Overall, in Fig. 10(c), six nodes have a total of 1828 new concepts and 25 modified concepts, as was shown for the major subject node ‘peptide’ in Fig. 7. This illustrates that a DWAT, for two secondary subject taxonomies of the same major subject, can provide more detailed information for changes of the major subject node. Users can similarly further drill down, if necessary, into each of the secondary subject nodes, to obtain further information about their evolution, by deriving a DWAT between the corresponding tertiary subject taxonomies of the same secondary subject in two releases. This way evolution of an ontology can be studied in the desired level of detail.

## Discussion

In our previous paper [18], we developed the child-of-based layout of an aggregate taxonomy, as example, using release 1.0.108 of CIDO which is the old release in this study. However, the WAT with 25 nodes in [18] is different from Fig. 5 in this paper. The reason is that the former one is derived from the (domain $\cup$ restriction)-defined partial-area taxonomy [28] while Fig. 5 is derived from the restriction-defined partial-area taxonomy [28]. The derivation methodology of these two taxonomies is the same but the way to define an area is based on different relationships in an ontology. Ochs et al. [28] discussed the granularity of domain-defined taxonomies, restriction-defined taxonomies, and (domain $\cup$ restriction)-defined taxonomies.

Object properties (relationship) in OWL can have explicitly defined domains and can be used in restrictions on classes (concepts). The major difference of these two usages of object properties is that a restriction is local (i.e., on specific concepts) while domain specification (i.e., a hierarchy) is global. The object property difference results in the difference of the two WATs mentioned before. The 25 nodes of the (domain $\cup$ restriction)-defined partial-area taxonomy in [18] include some nodes rooted at general concepts without much specific information like *entity*, *continuant*, and *occurrent*. Users need to drill down to see more information under each node rooted at such top-level concepts. In contrast, Fig. 5 is more informative where such top-level concepts are inside the root node ‘Thing’.

In general, one can generate a DWAT of any two releases of an ontology. However, there are two particular situations which are most informative about the evolution of an ontology. One situation is as we compare two WATs of two releases when the same parameter b is used for both. Clearly the number of nodes in the WAT of the later release will be larger than for the older release. By using the same parameter, such a comparison would emphasize the concepts which were added to CIDO between those two releases. In particular the DWAT will highlight new kinds of concepts which did not exist in the earlier version of CIDO, or that their weight (i.e., the number of descendant concepts) was below the parameter b. Such nodes in DWAT are highlighted in green. Of course, if the number of concepts of a given kind was reduced, by their deletion, below the parameter, then such kind of major subjects in the WAT of the old version may disappear from the WAT of the new version. Such a situation is highlighted in red in the DWAT. In addition, the DWAT shows in which of the major subjects, there was a large number of new concepts and in which only a few were added. Those changes are highlighted by the number of added concepts in the nodes of the DWAT highlighted in yellow.

Another most informative comparison happens when we have in the WATs of both releases approximately the same number of nodes. Such comparison emphasizes the same number of major subjects, in the two releases of CIDO, independent of the number of the concepts in the two releases. Of course, to obtain about the same number of nodes in the larger release, a higher parameter b is necessary for the WAT of this release. In the DWAT of such comparison we can expect to see more nodes highlighted in red, than in the previous DWAT, when the number of concepts of some kinds was larger than the smaller parameter b for the earlier release, but smaller than the larger parameter for the later release. On the other hand, we will see in the DWAT about the same number of nodes highlighted in green for new major subjects as the number of red-highlighted nodes, due to the addition of concepts of new kinds to CIDO or of kinds that had only a few concepts in the earlier release which did not warrant a status of a major subject. The reason is that since the number of nodes in both WATs is about the same, the number of introduced nodes and deleted nodes in DWAT must be about the same.

In the Results section we have demonstrated two DWATs, one for each of those particular situations. For this we compared the WAT of the June 2020 CIDO release containing 5,138 concepts with two WATs of the August 2022 release of CIDO containing 10,255 concepts. CIDO almost doubled in the number of concepts during this period. For the 2022 release, one WAT uses the same parameter as the WAT of the 2020 release. The second WAT has about the same number of nodes as the WAT of the 2020 release. Hence, we obtained two perspective views about the evolution of CIDO from 2020 to 2022.

For example, looking at the DWAT of Fig. 7 we see four red nodes which stopped being major subjects in the evolution of CIDO from 2020 to 2022, in spite of the growth of CIDO during this period. Two of these four nodes are ‘mesoderm-derived structure’ and ‘tissue’. Originally the concepts in the ‘mesoderm-derived structure’ and ‘tissue’ nodes were imported into CIDO from the Uberon ontology [29] using the tool OntoFox [30]. However, the CIDO curators realized that most of those concepts are not relevant for the COVID-19 disease. Thus, 27 mesoderm-derived structure concepts and 55 tissue concepts were deleted.

In contrast there are many more added major subjects highlighted with green in the DWAT. Since the later release has many more concepts it is expected to have many more added major subjects than deleted major subjects. Among the 15 added major subjects we find: ‘vaccine component’, ‘host-coronavirus interaction’, ‘AA variant in SARS-CoV-2 S protein’, ‘AA variant in SARS-CoV-2 ORF lab polyprotein product’, ‘COVID-19 diagnostic testing device’, ‘COVID-19 vaccine’ and its two children, ‘COVID-19 subunit vaccine’ and ‘COVID-19 RNA vaccine’. It is not surprising that these new major subjects were added later to the CIDO as they reflect more advanced issues which were explored later as the research into the COVID-19 disease advanced. For example, new concepts representing COVID-19 diagnostic testing devices, such as home testing device and polymerase chain reaction (PCR) testing kits [31, 32], and new concepts for different types of COVID-19 vaccine, such as Moderna COVID-19 vaccine in the node ‘COVID-19 RNA vaccine’, COVAX-19 [33] in the node ‘COVID-19 subunit vaccine’, and AdCOVID [34] in the node ‘COVID-19 vaccine’.

Most of the other new major subjects were related to chemicals. For example, the node ‘vaccine component’ has 32 concepts, of which 24 were added in the 2022 release. An example concept of vaccine component is *Advax vaccine adjuvant*, which has been used as the vaccine adjuvant for the COVID-19 vaccine called COVAX-19 [33]. The concepts in the two nodes ‘AA variant in SARS-CoV-2 S protein’ and ‘AA variant in SARS-CoV-2 ORF1ab polyprotein product’ were important to the presence of various coronavirus variants, such as Delta and Omicron strains. These coronavirus variants include various amino acid (AA) variants. For example, D614G is a SARS-CoV-2 AA variant that is located at the position 614 in the Wuhan reference strain S protein, and has been changed from D in the reference protein to the G residue [35]. The D614G mutation has significant impact on coronaviral pathogenesis, immune escape, transmission, and vaccine development [36].

The node ‘host-coronavirus interaction’ contains 425 new concepts, which were added to CIDO due to the importance of such interactions in the development of the COVID-19 disease. The example concept *SARS-CoV-2 S binding to human ACE2* plays an important role in the binding of coronavirus to human cells [37].

There are 17 nodes in the DWAT of Fig. 7, which are modified and are highlighted in yellow. This is an expected phenomenon, since the number of concepts in CIDO grew during this evolution. Nevertheless, we see some modified nodes for which their number of concepts decreased. For example, the number of concepts in the node ‘anatomical structure’ was reduced by 120 concepts (as marked in bold under the area name), mainly due to the deletion of 126 concepts with the same story as its two child nodes ‘mesoderm-derived structure’ and ‘tissue’ mentioned above. One of the two new concepts in this node is *renin-angiotensin system* (RAS), which is primarily responsible for blood pressure control. SARS-CoV-2 uses the angiotensin-converting enzyme-2 (ACE2) as the receptor for cell entry and thus directly interacts with RAS [38].

A noteworthy modified node in Fig. 7 is ‘peptide’ where 1853 concepts were added. The reason why so many peptide concepts were imported from Chemical Entities of Biological Interest (ChEBI) [39] in the later release of CIDO is that many medication ingredients, e.g., cyclosporin A and vancomycin, are proteins which are peptides. Another noteworthy peptide is *angiotensin*, a peptide hormone that causes vasoconstriction and increases blood pressure. The angiotensin-converting enzyme (ACE) and ACE2 actively participate in the metabolism of angiotensin. The binding of SARS-CoV-2 S protein to the host ACE2 significantly changes the angiotensin metabolism, leading to various outcomes [40].

The differences between the two DWATs in Figs. 7 and 9 are described in the Results section and are not repeated here. In general, Figs. 6 and 8 are demonstrating two summarization views of the same 2022 release. Those two WATs are of different refinement levels. The difference is caused by different values of b resulting in a meaningful difference in the number of major subjects. The refinement level is expressed in the difference about which nodes qualify as major subjects of the ontology. This difference is also expressed in the corresponding DWATs between each of the above two WATs and the WAT of the 2020 release. In this paper, we demonstrate the ability to derive DWAT between WATs of different number of nodes, as well as different values of the parameter b.

As shown in the Results section, the DWAT can extend to more detailed levels of secondary subject taxonomies. The DWAT can be a powerful mechanism enabling curators and users of ontologies follow the evolution of an ontology between releases, by providing a “big picture” of the differences. Without such a mechanism users of ontologies may “lose the forest for the trees.”

### Limitations

We note that the suggested DIFF framework is not recommended for small ontologies. For small ontologies of several hundreds of concepts, like the COVID-19 Infectious Disease Ontology having 486 concepts [10], their summarization by partial-area taxonomies [15] is small enough to be visualized. Hence, the previous Diff Partial-area Taxonomy technique [26] based on the partial-area summarization network is satisfactory for tracking the evolution of such small ontologies.

The case where the Diff Weighted Aggregate Taxonomy technique presented in this paper is required to track the evolution, is for large ontologies of the magnitude of thousands of concepts, e.g., CIDO with 10,255 concepts. Furthermore our technique can be applied to track the evolution of hierarchies of very large ontologies such as SNOMED CT [41], NCI Thesaurus (NCIt) [42], or Gene Ontology [43], which consist of up to hundreds of thousands of concepts. It is true that one can apply our technique for a whole ontology like SNOMED CT or NCIt. But due to the limitations of the number of nodes that humans can comprehend, the large and complex Diff network for such whole ontology will not enable humans to follow details of the evolution. Thus, for such large ontologies, it is recommended to apply the divide and conquer approach and track the evolution of each hierarchy of interest by itself. As a matter of fact, even some hierarchies of large ontologies are very large. For example, the *Clinical finding* hierarchy of SNOMED CT is larger than 100,000 concepts while its *Procedure* hierarchy has more than 50,000 concepts. In such cases one may want to track the evolution of subhierarchies, such as “Cardiovascular finding” subhierarchy of the *Clinical finding* hierarchy or “Procedure on cardiovascular system” of the *Procedure* hierarchy in SNOMED CT, or *Neoplasm* subhierarchy in the NCIt *Disease, Disorder or Finding* hierarchy.

These possibilities illustrate the flexibility of our technique to enable the adjustment of the granularity of the Diff Weighted Aggregate Taxonomy to the needs of each ontology or hierarchy of a large ontology. By calibrating the parameter *b* according to the size of the ontology or the hierarchy involved, users can pick at what granularity the evolution is displayed. The ability of picking a subhierarchy of an ontology for displaying the Diff Weighted Aggregate Taxonomy, enables users to concentrate on a portion of an ontology where extensive remodeling was performed, for example, to review the “Bacterial infectious disease” subhierarchy in the *Clinical finding* hierarchy of SNOMED CT which went through extensive remodeling by a curator of SNOMED CT [44–46].

## Conclusions

Curators and users of an ontology need to follow its evolution over time. Considering the size and complexity of an ontology, finding the differences between two releases of an ontology is a difficult and tedious challenge. It is necessary to summarize the differences between two releases to provide the "big picture" of the evolution in a compact way. In the paper we utilize the notion of a compact summarization network called Weighted Aggregate Partial-area Taxonomy (WAT) to introduce the new Diff Weighted Aggregate Taxonomy (DWAT) between two WATs of two releases of an ontology. A visualization of the DWAT is provided. Such visualization will enable curators and users of an otology to track its evolution. The DWAT is illustrated by comparing two releases of the Coronavirus Infectious Disease Ontology (CIDO), a fast-growing ontology to support research on treatments, medications, and vaccines for the COVID-19 disease. It is illustrated how the DWAT of CIDO provides insight into the evolution of CIDO.

## Data Availability

The datasets used and/or analysed during the current study are available from the corresponding author on reasonable request. The software OAF is available at https://saboc.njit.edu/software.php.

## Abbreviations

CIDO: Coronavirus Infectious Disease Ontology
SABOC: Structural Analysis of Biomedical Ontologies Center
OAF: Ontology Abstraction Framework
PAT: Partial-area taxonomy
DPAT: Diff Partial-area Taxonomy
WAT: Weighted Aggregate Partial-area Taxonomy
DWAT: Diff Weighted Aggregate Taxonomy
AA: Amino acid
RAS: Renin-angiotensin system
ACE2: Angiotensin-converting enzyme-2
ChEBI: Chemical Entities of Biological Interest
ACE: Angiotensin-converting enzyme
NCIt: NCI Thesaurus
SNOMED CT: SNOMED Clinical Terms
